# Ultra-fast photo-carrier relaxation in Mott insulators with short-range spin correlations

**DOI:** 10.1038/srep21235

**Published:** 2016-02-17

**Authors:** Martin Eckstein, Philipp Werner

**Affiliations:** 1 Max Planck Research Department for Structural Dynamics, University of Hamburg-CFEL, 22761 Hamburg, Germany; 2 Department of Physics, University of Fribourg, 1700 Fribourg, Switzerland

## Abstract

Ultra-fast spectroscopy can reveal the interplay of charges with low energy degrees of freedom, which underlies the rich physics of correlated materials. As a potential glue for superconductivity, spin fluctuations in Mott insulators are of particular interest. A theoretical description of the coupled spin and charge degrees of freedom is challenging, because magnetic order is often only short-lived and short-ranged. In this work we theoretically investigate how the spin-charge interactions influence the relaxation of a two-dimensional Mott-Hubbard insulator after photo-excitation. We use a nonequilibrium variant of the dynamical cluster approximation, which, in contrast to single-site dynamical mean-field theory, captures the effect of short-range correlations. The relaxation time is found to scale with the strength of the nearest-neighbor spin correlations, and can be 10–20 fs in the cuprates. Increasing the temperature or excitation density decreases the spin correlations and thus implies longer relaxation times. This may help to distinguish the effect of spin-fluctuations on the charge relaxation from the influence of other bosonic modes in the solid.

The interplay of charge and spin degrees of freedom in Mott insulators is a paradigm for emergent physics in strongly correlated electron systems. In dimensions *d* > 1, a carrier in an antiferromagnetically ordered background flips a spin in every hopping process, which leads to an intrinsically strong spin-charge interaction. This interaction prevails even in the paramagnetic phase, where spin correlations are short-ranged and short-lived, and it may play an important role in the formation of superconductivity. Spectroscopically, the spin-charge interaction is evidenced by narrow spin polaron peaks resulting from the binding of holes into string-states^1^,^2^,^3^,^4^,^5^, but these signatures become less pronounced in the absence of long-range order. Ultra-fast time-resolved experiments can provide an entirely different view on the interaction of charges with low-energy degrees of freedom, by tracking the time-evolution of photo-doped carriers. In Mott insulators, the relaxation after photo-doping, i.e., the insertion of mobile carriers with a short laser pulse, becomes highly non-trivial due to the strong interactions between electrons, spins and the lattice. The photo-excited metallic state decays within picoseconds, which is orders of magnitude faster than in semiconductors^6^,^7^,^8^,^9^,^10^,^11^. On even shorter timescales, optical measurements can unravel processes during the build-up of the metallic state. In the cuprate Nd_2_CuO_4_, Okamoto *et al*.^9^ found a rapid relaxation of the Drude peak in the optical conductivity within less than 40 fs, followed by the formation of a mid-gap absorption band which indicates the existence of polaronic quasiparticles. More recently, reflectivity measurements in the cuprate Bi_2_Sr_2_Y_0.08_Ca_0.92_Cu_2_O_8+*δ*_ showed the build-up of an effective electron-boson interaction on a timescale as fast as 16 fs^12^.

The strong spin-charge interaction is a natural candidate for the fastest energy relaxation process in Mott insulators with strong antiferromagnetic correlations. But can its effect be disentangled from other relaxation channels? A powerful strategy for analyzing time-resolved measurements assumes the coupling of charges to bosonic modes, whose spectrum can then be extracted from time-resolved experiments^13^,^14^,^15^,^16^. However, this procedure often does not fully reveal the microscopic nature of the respective modes. Additional insights could be gained if the system is driven far enough from equilibrium that the relevant bosonic modes are themselve affected by the excitation. In particular, the population of a harmonic oscillator mode has no upper bound, while the number of spin-flips in the system is intrinsically limited. This suggests that distinct signatures of the relaxation due to spin-flip scattering may be found by changing temperature, carrier doping, or laser fluence, since increasing these parameters makes the spin order more short ranged and short lived.

In this work we investigate the influence of short-range spin-fluctuations on the relaxation of photo-induced charges in extended paramagnetic Mott insulators. In nonequilibrium, the interaction of carriers with an antiferromagnetic background on a femtosecond timescale has been studied with exact diagonalization techniques^17^,^18^,^19^,^20^, spin-wave approximations^21^, and nonequilibrium dynamical mean-field theory^22^,^23^. A corresponding analysis of the paramagnetic phase, where spin-correlations are short-ranged and short-lived, has so far been restricted to exact diagonalization of small systems^12^. These simulations are nicely in agreement with the observed sub 20 fs relaxation times, but in small systems it is difficult in particular to systematically analyze different carrier numbers, and the effect of changing the excitation density. To study the dynamics of extended correlated systems, the nonequilibrium dynamical mean-field theory (DMFT) is in principle a powerful theoretical approach^24^,^25^. However, while DMFT has been used extensively to study the antiferromagnetic phase with long-range order^22^,^23^,^26^,^27^,^28^, it neglects short-range spin-correlations in the paramagnetic phase. Relaxation processes in DMFT are thus restricted to electron-electron scattering, leading to a very slow or absent redistribution of spectral weight^29^,^30^,^31^, which may correctly describe systems at high temperature and high fluence, in which spin-correlations have been melted away (see below). To overcome these limitations, we go beyond DMFT and implement a nonequilibrium version of the dynamical-cluster approximation (DCA)^32^, which maps a lattice problem to a small cluster embedded in a self-consistent bath, and thus allows us to incorporate the effect of short-range correlations on the real-time evolution of an extended lattice system.

## Results

### Model and equilibrium properties

To study the interaction of photo-doped carriers with spin-fluctuations we consider the Hubbard model on a two-dimensional square lattice
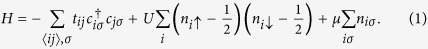


Here the operators *c*_*iσ*_ create an electron with spin *σ* at lattice site *i*, *U* is the on-site interaction, and *t*_*ij*_ is the nearest neighbor hopping. We measure energy in units of t_*_ 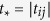, time in units of 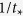
t_*_(*ħ* = 1). Taking typical parameters for a cuprate Mott insulator (*U* = 3 eV, t_*_ = 0.25 eV, i.e., *U*/bandwidth = 1.5), the unit of time is 2.6 fs, and temperature is measured in units of t_*_/*k*_*B*_ = 2900 K. Note that a quantitative modeling of both photo-doped holes and electrons in cuprates would require not only a next nearest neighbor hopping term (which is straightforward to incorporate), but also a multi-band model if particles are excited across the charge transfer gap. However, the particle-hole symmetric model (1) can be expected to capture the qualitative effects of carrier relaxation in the presence of short-range spin fluctuations.

**Figure 1:**
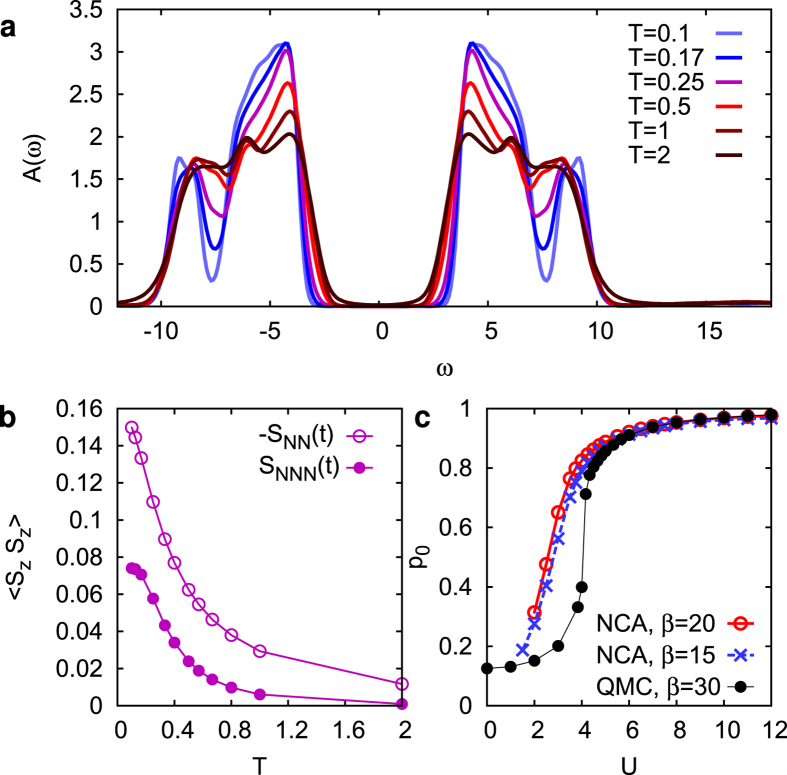
Equilibrium DCA results. (**a**) Temperature dependent spectral function in the half-filled insulator for *U* = 12. (**b**) Nearest neighbor (NN) and next-nearest neighbor (NNN) spin-correlations in the impurity model. (**c**) Occupation probability *p*_0_ of the plaquette-singlet state (see text); QMC data are taken from ref. 36.

To solve the dynamics in the Hubbard model we employ the nonequilibrium formulation of DCA, with a cluster of 2 × 2 sites, using the lowest order self-consistent hybridization expansion (non-crossing approximation, NCA) as a cluster impurity solver. Technically, this approach requires a multi-orbital generalization of the NCA, and thus the solution of a large number of coupled real-time Dyson equations, for propagators representing each symmetry sector of the local Hilbert space. Details of the numerical implementation are given in the method section. In equilibrium, the NCA has been used as an impurity solver for DCA^33^,^34^,^35^ even in the superconducting and pseudo-gap phase. As NCA is based on an expansion around the isolated cluster, the results are expected to be most reliable in the Mott phase. This is confirmed in Fig. 1c, which shows a comparison to continuous-time Quantum Monte Carlo results^36^ for the probability *p*_0_ to find the system in the “plaquette singlet state” (i.e., the ground state of the isolated cluster), which reflects the crossover from metallic to insulating behavior.

In the following we focus on the insulating phase at *U* = 12. As a measure for the short-range spin correlations, we compute the spin correlations in the 2 × 2 cluster model,
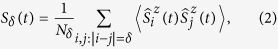
where *S*_*i*_ is the spin operator at site *i*, and 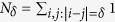 is the number of sites with distance *δ*. In equilibrium, nearest and next nearest neighbor spin correlations, 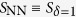 and 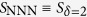, clearly show a crossover to strong antiferromagnetic correlations below temperature *T* ≈ 0.5 (Fig. 1b). At high temperature, the solution becomes similar to single-site DMFT^36^, which is also evident in the spectral function (Fig. 1a). With increasing temperature, the gap slightly shrinks, and the sub-structure of the Hubbard bands is washed out. We note that the emergence of sub-structure in the Hubbard bands with the build-up of short range correlations is also observed with Quantum Monte Carlo^36^ and exact-diagonalization^37^ as an impurity solver. However, spectral properties at high energies are hard to infer from imaginary time data or from a discrete bath, so that a direct comparison to NCA is not possible.

### Relaxation in the undoped Mott insulator

We now simulate the time evolution of the insulating phase at *U* = 12 after excitation with a laser pump at frequency Ω = 16, which populates states in the upper Hubbard band. The laser excitation is modeled by a few-cycle electric field pulse of the form 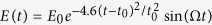 with a frequency Ω comparable to the gap, polarized along the body-diagonal of the lattice 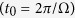. The electric field ***E***(*t*) of the pump-laser pulse is incorporated into the Hamiltonian (1) by a Peierls phase (see methods).

**Figure 2:**
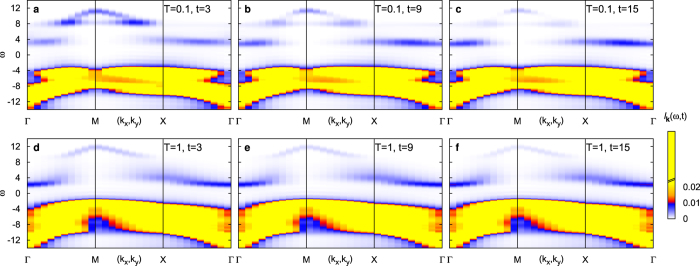
Momentum- and time-resolved photo-emission spectrum *I*_***k***_(*ω*, *t*). The results are for an undoped Mott insulator with *U* = 12 at low temperature *T* = 0.1 (panels (**a**–**c**)) and high temperature *T* = 1 (panels (**d**–**f**)), for three different times. The momentum ***k*** is varied along a path 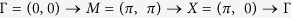 though the Brillouin zone. The pump-pulse amplitude is *E*_0_ = 6, corresponding to an excitation density *N*_ex_ ≈ 0.9%. The color map is constant (yellow) for all intensities *I* > 0.02; a linear color scale cannot resolve the very different weight of the lower Hubbard band at *ω* < 0 and the photo-induced occupations in the upper Hubbard band at *ω* > 0.

Figure 2 shows the time and angular-resolved photo-emission spectrum (ARPES) ***I***_***k***_(*ω*, *t*) for two different temperatures. The ARPES intensity is computed from the momentum-resolved Green’s function ***G***_***k***_, using a Gaussian probe pulse with and time resolution Δ*t* = 3, i.e., energy resolution 1/Δ*t*^38^ (see methods). The spectrum of the Mott insulator before excitation features a broad and occupied lower Hubbard band. Immediately after photo-excitation, some occupation appears within the upper Hubbard band (Fig. 2a,d). The photo-induced occupation covers the full energy range due to the broad-band power spectrum of the pump pulse. In the subsequent evolution, the occupation is cascading from the highest energies, located around the point ***M*** = (*π*, *π*) in the Brillouin zone, down to the center of the Brillouin zone (Γ). There are two marked differences between the dynamics at high temperature *T* = 1 (Fig. 2d–f) and low temperature *T* = 0.1 (Fig. 2a–c). First, (i), the spectral features at *T* = 0.1 show less energy dispersion than at *T* = 1. Although the resolution of a DCA with a 2 × 2 cluster is limited, this indicates a stronger localization of charges due to the more pronounced local spin order. Interestingly, this stronger localization is observed already at the earliest times. This indicates that the dressing of charges with fluctuations happens within a few hopping times (during the pulse), reminiscent of the formation of lattice polarons in the regime at very high coupling^39^. More importantly, (ii), the relaxation dynamics at low temperature appears to be considerably faster. In the following we will analyze the temperature dependence of the relaxation time more systematically in terms of the angular-integrated spectrum, and show that it reflects the spin fluctuations.

### Temperature-dependent relaxation times

**Figure 3:**
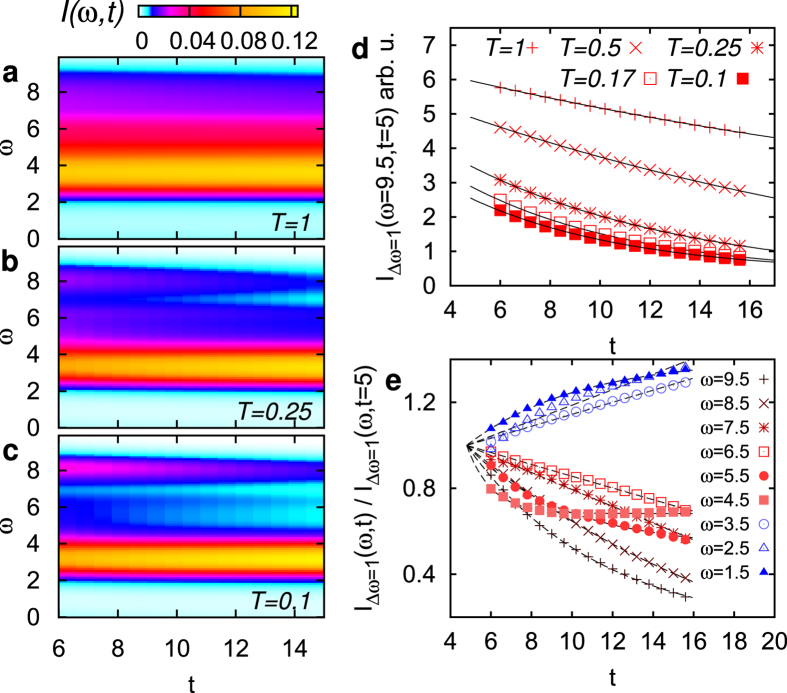
Time dependent energy distribution of the photo-carriers. (**a**–**c**) time-dependent photo-emission spectrum in the upper Hubbard band, for three different temperatures. (Half-filled insulator at *U* = 12, amplitude *E*_0_ = 6, *N*_ex_ ≈ 0.9%). (**d**) PES weight integrated in the high-energy window 9 ≤ *ω* ≤ 10 for various temperatures. The solid lines are exponential fits. (**e**) Frequency dependence of the relaxation for low temperatures, *T* = 0.1. These curves are normalized at *t* = 5.

Figure 3a–c shows the time and energy-resolved photo-emission spectrum (PES) ***I***(*ω*, *t*) for three different temperatures. The angular integrated PES is computed from the local (momentum-averaged) Green’s function, using the same Gaussian probe pulse as in Fig. 2. The excitation density *N*_ex_ can be defined by the integrated photoemission weight in the upper Hubbard band, when the total weight 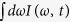 is normalized to the particle density. For *U* = 12 and the pump with Ω = 16 we find a roughly constant absorption coefficient 
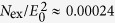
 up to 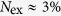. During the timescale of the simulation, we observe a small increase of the total weight in the upper Hubbard band (up to 3% of *N*_ex_). This indicates carrier multiplication processes which can be important if the Hubbard gap is comparable to the bandwidth^40^. For the following discussion these effects are not dominant and will not be studied systematically.

**Figure 4:**
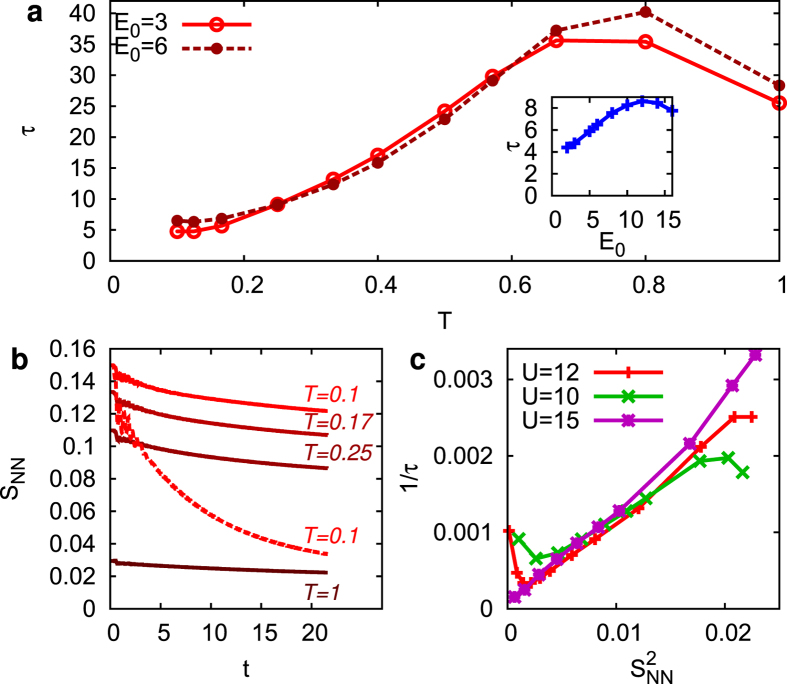
Relationship between relaxation times and spin correlations. (**a**) Decay-time of high-energy PES at *U* = 12 as a function of temperature at two fluences; the relaxation time is obtained from the exponential fits shown in Fig. 3d. Inset: Dependence of *τ* for temperature *T* = 0.1 on the amplitude *E*_0_. (**b**) Time-dependence of *S*_NN_ after excitation with low fluence (*E*_0_ = 6, *N*_ex_ ≈ 0.9% solid lines), and high fluence (*E*_0_ = 14, *N*_ex_ ≈ 4.5%, dashed line). (**c**) Relaxation rate 1/*τ* in the low fluence regime (*E*_0_ = 3, *N*_ex_ ≈ 0.2%) computed for various temperatures and plotted as a function of the equilibrium value 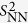. For each value of *U*, relaxation times have beed obtained from the time-dependent weight 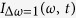 around the upper band edge (*U* = 10: *ω* = 8.5, pump frequency Ω = 13; *U* = 12: *ω* = 9.5, Ω = 16; *U* = 15: *ω* = 10.75, Ω = 18).

We first focus on the low-fluence regime (excitation density 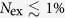), and discuss the fluence dependence later. On the timescale of the simulation, the redistribution of spectral weight from high to low frequencies is clearly more pronounced at low temperature (*T* = 0.1, Fig. 3c) as compared to high temperature (*T* = 1, Fig. 3a). To analyze the redistribution of weight within the upper band, we integrate the PES over a given energy interval Δ*ω*, 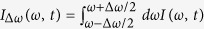. A decay of high energy doublons implies a decrease and increase of spectral weight at high end low energies, respectively, while at intermediate energies the spectral weight can transiently either increase or decrease (Fig. 3e). To see the main role of various scattering channels we first focus on the decay of the high energy states, which shows an exponential time-dependence even on the short times simulated here (Fig. 3d). The curves are fitted with a single-exponential decay 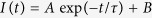, and the relaxation rates are plotted in Fig. 4a. The relaxation rate increases with temperature with a maximum at very high temperatures.

As mentioned in the introduction, a possible candidate for relaxation is the scattering with short-ranged spin fluctuations. The disturbance of the short-range spin correlations and associated transfer of energy to the spin sector can be seen on a qualitative level through a decrease of the nearest-neighbor spin correlations *S*_NN_(*t*) (Fig. 4b), which becomes more prominent with increasing fluence. For a carrier in a static antiferromagnetic background, it is possible to estimate a maximum energy transfer rate from mobile carriers to the spin sector^23^: For each hopping the average number of spin-flips against the antiferromagnetic exchange *J* is proportional to the order parameter *m*, and hence the maximal spin-flip rate 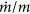 is proportional to a hopping time. Consequently, the maximum rate of change of the spin energy *Jm*^2^ is proportional to *m*^2^. Motivated by this argument, we plot the temperature-dependent relaxation rate 1/*τ* of the high energy part of the PES against 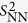 (Fig. 4c). We observe a linear scaling in the intermediate temperature range, which leads to a main result of our work, namely that the inverse relaxation time can be taken as a measure of spin-correlations in the paramagnetic phase. (In agreement with this, a single-site DMFT simulation, which does not capture non-local spin fluctuations at all, would produce almost no net redistribution of spectral weight from high to low energies on the timescale of Fig. 2). The saturation and maximum of the relaxation time at large temperatures (small *S*_NN_) can be interpreted as a deviation from the simple spin picture. In agreement with this, we observe at these temperatures a slight filling-in of the gap (see Fig. 1a), and the saturation effect is shifted to larger temperatures as the interaction is increased from *U* = 10 to *U* = 15 (Fig. 4c).

**Figure 5:**
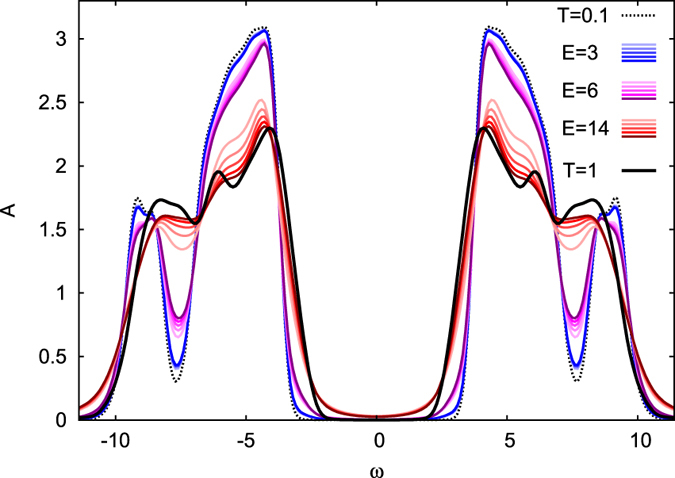
Fluence dependence of the time-resolved spectral function *A*(*ω*, *t*). The figure shows results for a half-filled insulator at *U* = 12, *β* = 10 for three fluences (*E*_0_ = 3, *N*_ex_ ≈ 0.2%; *E*_0_ = 6, *N*_ex_ ≈ 0.9%; *E*_0_ = 14, *N*_ex_ ≈ 4.5%) and various times (t = 6, 8.4, 10.6, 12, 14.4 from light to dark colors). The thick black solid line is the spectral function at high temperature *T* = 1, the dashed line is the spectral function at he initial temperature *T* = 0.1. For high fluences, the spectrum around the Hubbard bands becomes more similar to the high-temperature spectrum at half-filling. However, the spectrum does not indicate thermalization. The larger number of mobile carriers of the photo-induced state is reflected in the accumulation of spectral weight in the gap.

Another important feature is the dependence of the relaxation time on the excitation density. For large fluences we observe a rapid decrease of spin-correlations after the pulse (Fig. 4b) due to spin-flips by photo-induced doublons and holes. Consistent with this observation and the dependence of the relaxation time on spin-correlations, we observe an increase of *τ* with fluence (Fig. 4a, inset). (With the melting of short-range spin-fluctuations, also the spectral function around the Hubbard peaks becomes similar to the high-temperature spectral function, cf. Fig. 5). An order of magnitude estimate shows that a melting of short-range spin correlations at the given excitation density can be expected: For an excitation density of *N*_ex_ = 4.5% (*E*_0_ = 14) and a bandwidth of *W* = 10, photo-excitation can provide an energy of 0.5 per spin, which is comparable to the temperature scale set by the exchange energy, on which spin correlations decrease (cf. Fig. 1b). In contrast, the heating of lattice phonons in pump-probe experiments is usually much *less* than that of the electrons, due to their larger heat capacity. Hence the pronounced fluence dependence of the relaxation behavior due to the disordering of spins may help to distinguish spin fluctuations from other relaxation processes, such as phonons.

While we have so far looked at the relaxation of the PES *at high energies*, it is worthwhile to briefly discuss how the measured relaxation time depends on the observable. Within the simulated time window the time-evolution of the weight *I*(*ω*, *t*) is not an exponential relaxation for all frequencies (Fig. 3). At intermediate frequencies, e.g., weight is scattered both into and out of the states, leading to a non-monotonous time-evolution. For the same reason, also the time-evolution of averaged quantities such as the kinetic energy is not purely exponential, although the relaxation of these quantities also reflects the slow-down with increasing temperature.

### Doped Mott insulator

**Figure 6:**
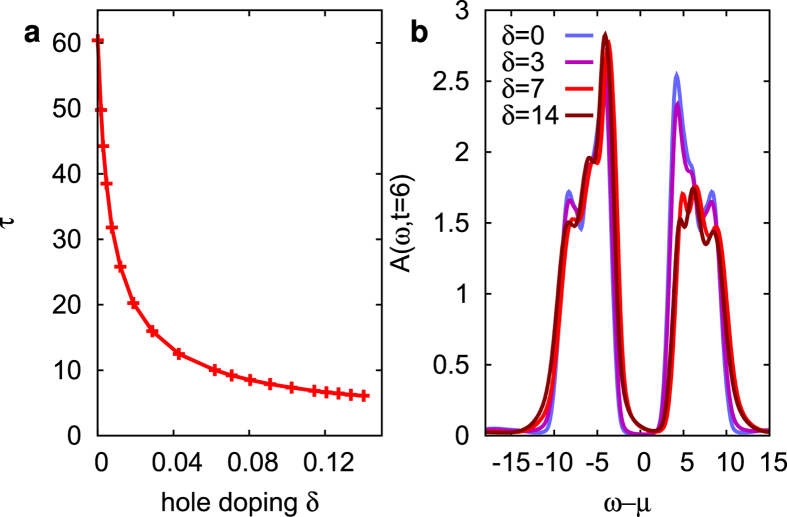
Influence of chemical doping on the relaxation time. (**a**) Relaxation time *τ* as a function of hole doping *δ* for *U* = 12, temperature *T* = 0.5, and fluence *E*_0_ = 3. The relaxation time is measured by an exponential fit to 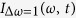 in the high-energy regime *ω* − *μ* = 9.5. (**b**) Corresponding spectral functions.

Finally, we briefly discuss the effect of doping on the relaxation rates. At finite hole doping *δ* one would enter the pseudo-gap or superconducting regime at low temperature, where a simple approximation like NCA becomes less reliable. However, an important qualitative effect of doping can already be seen at high-temperature: Fig. 6 shows that the high-energy relaxation time rapidly decreases with doping, to a few inverse hoppings. This is opposite to the effect of spin-correlations, since spin-correlations generally get reduced with increasing *δ*. The reason for this doping evolution is that for finite *δ*, additional mobile carriers provide an additional relaxation channel, in which a large number of chemically-doped carriers with low kinetic energy exchange energy with fewer photo-doped carriers.

## Conclusion

In conclusion, we have used the nonequilibrium extension of DCA to study the effect of non-local spin correlations in the paramagnetic Mott phase of the Hubbard model on the relaxation of photo-doped carriers. Our results show that the relaxation times of high-energy photo-doped carriers, measured by the decay of the photo-emission signal close to the upper edge of the Hubbard band, reflect the short-range spin correlations in the paramagnetic phase. Taking parameters appropriate for cuprates, the relaxation times range from ≈10–20 fs for low temperatures to ≈200 fs when spin correlations are suppressed. (Even for the lowest temperatures studied here, intra-cluster spin correlation decay rapidly within the cluster, which justifies the approximation of the 2 × 2 cluster. At lower temperature, spin correlations range over many lattice sites, and one may expect even shorter relaxation times.) At large fluence the transfer of energy from the electrons to the spins can substantially decrease the short-range fluctuations and thus quench the ultra-fast spin-charge relaxation channel. This shows that the fluence dependence can help to distinguish the relaxation due to spin fluctuations from other bosonic modes in an experiment. In future studies, it will be interesting to resolve the momentum and time dependent scattering at lower energies (which requires slightly longer simulation times), to see how not only nearest-neighbor spin correlations, but more generally the full momentum and frequency dependence of spin fluctuations may be obtained from the time-resolved photoemission (or two-photon photoemission) spectrum. More generally, our results show that short-range spin correlations act as a dissipative environment for mobile carriers, which may also be probed in cold atom experiments.

## Methods

### Nonequilibrium DCA

To solve the dynamics in the Hubbard model (1) we employ the nonequilibrium formulation of DCA. As for nonequilibrium DMFT^24^,^25^, the generalization of DCA to the time-domain can be done on a formal level by extending the theory from a Matsubara formalism to a Keldysh formalism^41^, and we restrict the discussion to issues relevant for the implementation of the approach in the strong-coupling regime. (For an introduction to the Keldysh formalism, and explanations concerning the notation for Keldysh Green’s functions, self-energies and Dyson equations, the reader is referred to ref. 25). The Brillouin zone of the lattice is divided into ***L***_*c*_ patches ***P***_***K***_, which in our case will be the patches around the high-symmetry points 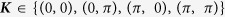. The lattice self-energy is approximated by a coarse-grained function 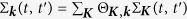 which is piecewise constant, i.e., 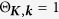 if 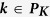 and 0 otherwise. The values 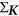 are obtained from an effective cluster model that consists of 2 × 2 = ***L***_*c*_ sites embedded in a self-consistent bath. Formulated in momentum space, the action on the Keldysh contour is given by
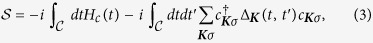

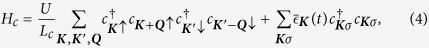
where Δ_***K***_ is the hybridization with the bath, and 
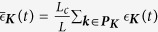
 is the patch-averaged dispersion (which is time-dependent in the presence of electric fields). From the cluster model we compute the patch Green’s functions 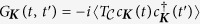 and the patch self-energy 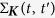 which are related by the Dyson equation 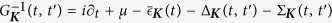. Momentum-dependent lattice Green’s functions are computed with the coarse-grained self-energy, 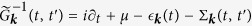, and the self-consistency is closed by the condition 
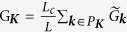
.

### Multi-orbital NCA

We use the lowest order self-consistent hybridization expansion (non-crossing approximation, NCA) to compute the Green’s function *G*_***K***_ from the action (3). NCA is a diagrammatic expansion around the atomic limit (Δ_***K***_ = 0), which can be formulated in terms of pseudo-particles representing the many-body eigenstates 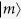 of the isolated cluster. The diagrammatic rules for NCA have been described in detail in ref. 42. For a general multi-orbital case, the pseudo-particle Green’s functions 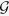 and self-energies are matrices with flavor indices *m*, so that the solution of real-time Dyson equations for these matrix-valued two-time Green’s functions can become numerically challenging. (The effort scales like 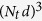, where *N*_*t*_ is the number of elementary time-steps, and *d* is the flavor-matrix dimension.) Taking into account quantum numbers 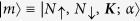 (particle numbers 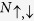, total momentum ***K***), the pseudo-particle Green’s functions are block diagonal, 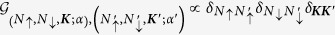, and the largest matrix dimension *d*_max_ is 12 (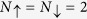, ***K*** = 0). The exponential scaling of *d*_max_ with *L*_*c*_ currently restricts the implementation of real-time NCA to the 2 × 2 cluster used here, which should be sufficient as a minimal model to capture short-range spin-fluctuations in an antiferromagnet. For the numerical solution we use a hybrid parallelized implementation, where Green’s functions for different quantum numbers are distributed over several compute nodes, while the solution of the pseudo-particle Dyson equations uses a shared memory parallelization.

### Electric fields and time-resolved photoemission spectrum

The electric field ***E***(*t*) of the pump-laser pulse is incorporated into the Hamiltonian (1) by a Peierls phase, i.e., we choose *t*_ij_ = t_*_ 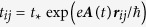, where ***r***_*ij*_ is the vector pointing from site *j* to *i*, and ***A***(*t*) is the vector potential from which the electric field is obtained by 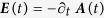. The field is measured in units of t_*_/*ea*, where *a* is the lattice spacing.

Photoemission spectra *I*(*ω*, *t*) are computed from the Green’s function using a Gaussian probe pulse 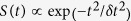 with and time resolution Δ*t* (i.e., energy resolution 1/Δ*t*)^38^, using the expression
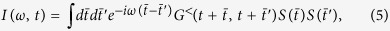
where *G* is the local Green’s function for the computation of the momentum-integrated spectrum. For the computation of the momentum-resolved spectrum *I*_***k***_(*ω*, *t*), we use the momentum-resolved Green’s function *G*_***k***_ in Eq. (5). The temporal overlap of the pump laser field and the probe envelope is small for the results presented in the manuscript, so that this description is gauge invariant^43^. The momentum-resolved Green’s function is obtained by interpolating the patch-dependent functions 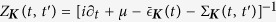 between the Brillouin zone patches, and then solving the Dyson equation 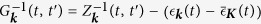.

## Additional Information

**How to cite this article**: Eckstein, M. and Werner, P. Ultra-fast photo-carrier relaxation in Mott insulators with short-range spin correlations. *Sci. Rep.* **6**, 21235; doi: 10.1038/srep21235 (2016).
